# Supplementary material for: Effect of Aging on the Immune Response to Core Vaccines in Senior and Geriatric Dogs
Source: Vet Sci. 2023 Jun 23;10(7):412. doi: 10.3390/vetsci10070412 (PMC10385316; doi:10.3390/vetsci10070412)

Table S1 - Dog's physiological age in human years based on its size [12]

	DOG'S PHYSIOLOGICAL AGE IN HUMAN YEARS			
	DOG'S SIZE			
AGE (years)	≤9 kg	10-22 kg	23-41 kg	>41 kg
1	15	15	15	15
2	24	24	24	24
3	28	28	30	32
4	32	33	35	37
5	36	37	40	42
6	40	42	45	49
7	44	47	50	56
8	48	51	55	64
9	52	56	61	71
10	56	60	66	78
11	60	65	72	86
12	64	69	77	93
13	68	74	82	101
14	72	78	88	108
15	76	83	93	115
16	80	87	99	123
17	84	92	104	--
18	88	96	109	--
19	92	101	115	--
20	96	105	120	--

Table S2 - VacciCheck: correspondence between S scale units and antibody titers, sensitivity, and specificity for Canine Parvovirus type 2 (CPV-2), Canine Distemper Virus (CDV), and Canine Adenovirus type 1 (CAdV-1)

	CPV-2 (%)	CDV (%)	CAdV-1 (%)
S0	<1:20	<1:8	<1:4
S1	1:20	1:8	1:4
S2	1:40	1:16	1:8
**S3 (threshold)**	**1:80**	**1:32**	**1:16**
S4	1:160	1:64	1:32
S5	1:320	1:128	1:64
S6	1:640	1:256	1:128
>S6	>1:640	>1:256	>1:128
Sensitivity (%)	*88*	*100*	*94*
Specificity (%)	*100*	*92*	*93*

Table S3 - Classification of protection categories for Canine Parvovirus type 2 (CPV-2), Canine Distemper Virus (CDV), and Canine Adenovirus type 1 (CAdV-1) based on antibody titers of VacciCheck

**Categories**	**CPV-2**	**CDV**	**CAdV-1**
Unprotected	<1:20	<1:8	<1:4
Weak Positive	≥1:20 to <1:80	≥1:8 to <1:32	≥1:4 to <1:16
Medium Positive	≥1:80*-<1:160	≥1:32*-<1:64	≥1:16*-<1:32
High Positive	≥1:160	≥1:64	≥1:32

* Titers with asterisk represent threshold values

Figure S1 Specific antibody titers for Canine Parvovirus type 2 (CPV-2), Canine Distemper Virus (CDV), and Canine Adenovirus type 1 (CAdV-1) in the 350 ageing dogs (titers with asterisk (*) represent the threshold values)

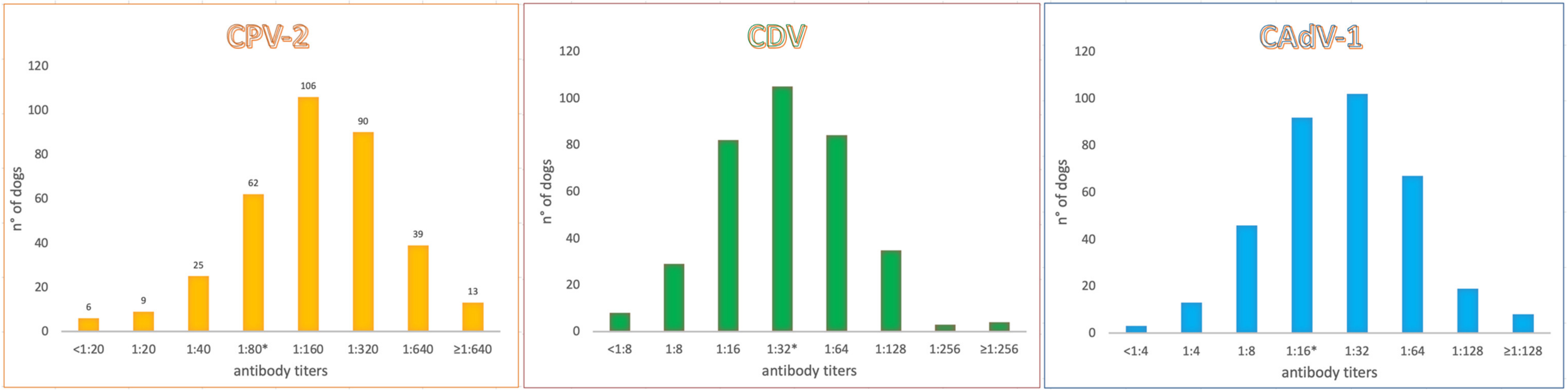

Supplement: Supplementary file 1 [file vetsci-10-00412-s001.zip › vetsci-2426217-supplementary.pdf]
